# Barriers and facilitators to neurodevelopmental follow-up among at-risk newborns across Washington State

**DOI:** 10.21203/rs.3.rs-9475637/v1

**Published:** 2026-05-20

**Authors:** Sara K. Neches, Megan Woods, Krystle Perez, Kendell German, Ulrike Mietzsch, John Feltner, Niranjana Natarajan, Cindy Trevino, Rebecca Penders, Sharilyn Emhoff, Bat-Sheva Stein, Sandra Juul, Brent R. Collett, Gregory C. Valentine

**Affiliations:** 1. University of Washington School of Medicine, Seattle, Washington; 2. Seattle Children’s Research Institute (SCRI), Seattle, Washington; 3. Washington State Perinatal Collaborative, Washington Department of Health; 4. Providence Inland Northwest Washington (INWA), Spokane, Washington; 5. Baylor College of Medicine, Department of Obstetrics & Gynecology, Houston, Texas

## Abstract

**Objective:**

Identify barriers and facilitators to neurodevelopmental follow-up (NDFU) for high-risk neonates in Washington (WA).

**Study Design:**

Cross-sectional, mixed-methods study of NDFU centers and neonatal intensive care units (NICUs) across WA to characterize NDFU referral and scheduling practices and identify barriers and facilitators to NDFU attendance.

**Results:**

Response rates were 84% for NDFU centers and 94% for NICUs. Barriers to NDFU attendance included logistical limitations (n=10, 63%) and scheduling processes (n=10, 63%), while facilitators included provider relationships and communication (n=8, 50%) and community-based access (n=8, 50%). No NICU respondent reported receiving any post-discharge communication from NDFU centers regarding patient developmental outcomes.

**Conclusion:**

Integrating anticipatory guidance and family education before NICU discharge with home-based services and telehealth may improve access to essential developmental follow-up care in this high-risk population. Interventions to increase NDFU attendance should prioritize family-centered communication, reduced logistical burdens, and flexible models of care delivery.

## Introduction

Although advances in neonatal care have significantly increased survival (1–3), the risk of long-term neurodevelopmental impairment (NDI) remains high for preterm infants and those affected by conditions such as neonatal encephalopathy, congenital anomalies, infections, and *in utero* illicit drug exposure (4,5) (6–8). The American Academy of Pediatrics (AAP) recommends neurodevelopmental follow-up (NDFU) for all high-risk infants to optimize early detection of delays (9–12).

In the United States, two complementary systems offer access to NDFU services (13,14). NDFU clinics are typically tertiary hospital-based, staffed by multidisciplinary teams, and focus on longitudinal surveillance and identification of NDI through standardized assessments and interdisciplinary evaluation. These clinics may also provide medical follow-up, nutrition and feeding support, mental health services, and physical or occupational therapy(13,15,16). Early Intervention (EI) programs, mandated under Part C of the Individuals with Disabilities Education Act (17), offer family-centered therapeutic services in the home or community for children under three years of age who have or are at risk for developmental delays, at no or limited cost to families (18). EI programs are typically staffed by physical, occupational, and speech therapists who assess infants and deliver early intervention services as needed (18). Together, NDFU clinics and EI programs form complementary systems of NDFU that support early detection of developmental delays and facilitate timely, individualized interventions to improve outcomes for high-risk infants (13,14,19).

EI services facilitated by NDFU centers have positive effects on motor and cognitive development in infancy with cognitive gains lasting into preschool (20,21). Despite these benefits, participation in NDFU remains challenging, with up to two-thirds of high-risk infants lost to follow-up by two years of age (22,23). Attrition is complex and multifaceted, influenced by interconnected medical, family, sociodemographic, and systemic, and sociopolitical factors(23–27). Additionally, differences in institutional practices and community resources lead to variations in follow-up rates, family counseling, and access to care, highlighting an important need to elucidate and overcome barriers to care. However, to determine best practices to promote higher NDFU rates by families of high-risk children, identification of the barriers and facilitators to NDFU care is a critical first step.

In partnership with the Washington State Department of Health and the Washington State Perinatal Collaborative, we sought to address this critical gap by identifying barriers that prevent and facilitators that enhance families’ attendance at NDFU care by conducting a statewide assessment of neurodevelopmental follow-up services throughout Washington state. Specifically, we examined referral, scheduling, and developmental screening practices across Level III and IV NICUs and NDFU centers, as well as the level of communication and collaboration between referring NICUs and neurodevelopmental programs, with the overall aim of informing strategies to promote equitable access to comprehensive neurodevelopmental care.

## Materials, Subjects and Methods

### Study design

Mixed-methods surveys, including both quantitative and qualitative questions, were distributed to providers at 19 NDFU centers (e.g., EI programs and ND clinics) and 16 regional NICUs across Washington State between September and November 2024. The surveys and study protocol were explicitly designed for providers of neonatal and NDFU care; no patients or direct patient data were obtained for this study. The study protocol was reviewed by the University of Washington Institutional Review Board and was determined to be exempt from review.

### Survey Development

Two online surveys were developed and reviewed by the study investigators using Research Electronic Data Capture (REDCap), a secure electronic data capture tool (28). A 27-item survey (Supplemental File A) was distributed to clinical directors at regional Level III and Level IV NICUs to capture information about NDFU referral and scheduling practices, how information on infant neurodevelopment is shared with both families and the NDFU programs to which they are referred, and to assess the developmental screening tools utilized in participating NICUs. A 43-item survey (Supplemental File B) was distributed to program directors of NDFU centers across Washington State, exploring the demographics of patients seen in different types of NDFU centers, funding sources, staffing models, scheduling practices, no-show rates, visit cadence, use of telehealth, types of assessments administered, methods of communication with referring providers, and perceived barriers and facilitators to NDFU attendance.

### Survey Distribution

A list of email contacts for medical and/or program directors for Level III and Level IV NICUs (11) and NDFU centers across Washington was made available through the Washington State Department of Health. Public survey links were distributed by email in September 2024. When needed, the surveys were redistributed up to three times through November 2024. An introduction to the surveys explained the rationale for data collection and estimated completion time. Consent was implied if the individual chose to complete the survey. While names were not requested, the surveys assessed respondents’ roles and organization names to assist with the categorization of responses.

### Statistical Analysis

Survey data were collected and stored in the University of Washington REDCap database and analyzed to explore referral and scheduling practices, as well as barriers and facilitators to NDFU care from providers’ perspectives. We used descriptive statistics, including frequencies, means, medians, standard deviations, and interquartile ranges. All data analyses were conducted using SAS Studio OnDemand for Academics (Cary, NC: SAS Institute Inc).

Free-text responses from providers at NDFU centers describing perceived barriers and facilitators were analyzed using inductive qualitative content analysis. Responses were imported into NVivo (QSR International) to facilitate data organization and coding. MW performed line-by-line coding using an inductive approach to generate initial codes directly from the data. Codes were iteratively refined and organized into higher-order themes based on the mechanism by which each barrier or facilitator related to NDFU attendance. When codes overlapped conceptually, they were categorized based on their predominant mechanism of influence on attendance. No prespecified frequency threshold was used to create a separate theme; instead, themes were defined based on distinct differences in how barriers or facilitators influenced attendance. SN reviewed the coding framework and thematic organization to ensure consistency, refine code definitions, and resolve discrepancies through discussion. Thematic frequencies were calculated at the respondent level to describe the relative prominence of each category across NDFU centers.

## Results

### Provider characteristics

**Figure 1:**
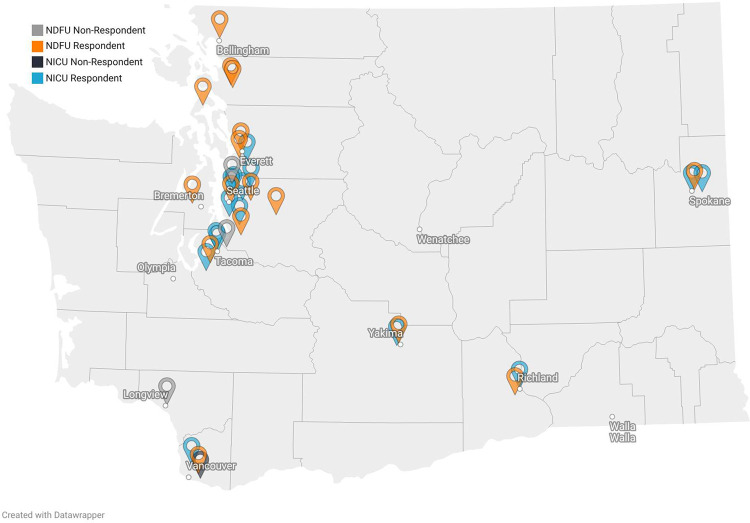
Geographic Distribution of Survey Respondents and Non-Respondents Across Washington State. The locations of all NICU and NDFU centers approached for surveys were identified using the Global Positioning System (GPS) and plotted on a map to indicate the type of center and respondent status.

A total of 15 providers representing eligible NICUs responded to the survey (94% response rate), including respondents from 10 of 11 Level III NICUs and all 5 Level IV NICUs across Washington State. Most NICU survey respondents held medical director roles, though staff knowledgeable in nursing, developmental care, and discharge coordination also had the opportunity to complete the survey. A total of 16 providers representing eligible NDFU centers completed the survey (84% response rate), including all 4 NDFU clinics in Washington State and 12 of 15 EI programs (Figure 1). Most respondents to the NDFU center survey held clinic director or program management roles (Table 1).

### NICU Survey

Only 4 NICU respondents (n=4/15, 26.7%) reported that their centers perform standardized neurodevelopmental assessments during the inpatient period; among those performing standardized neurodevelopmental assessments, only n=2 (50%) use the Hammersmith Neonatal Neurological Exam, n=3 (75%) use the General Movements Assessment, and n=1 (25%) use the Neonatal Neurobehavioral Scale. Over half of NICUs (n=8/15, 53.3%) utilize clinical feeding evaluations. Results of neurodevelopmental testing are primarily shared directly with the family by the therapist performing the exam (n=12, 80%), though this information may be shared by another NICU provider (n=4, 27%) or in a multidisciplinary care conference (n=4, 27%). The eligibility criteria for NDFU referral from the respondent NICUs included birth weight (defined as patient <1500 grams at birth, intrauterine growth restriction, or small for gestational age), gestational age (e.g., prematurity), hypoxic ischemic encephalopathy, seizures, history of surgery, and history of extracorporeal membrane oxygenation (ECMO) (Table 1). Most referrals for neonatal follow-up and early intervention are placed by the medical team (n=9, 60% and n=7, 47% respectively), though other providers play a significant role in referral placement, including discharge coordinators, social workers, and inpatient infant therapy team members. NDFU appointment scheduling was completed by a range of individuals, including discharge coordinators, medical providers, neurodevelopmental clinic coordinators, and therapy team members, while parents mostly complete EI scheduling.

### Neurodevelopmental Follow-Up and Early Intervention Survey

Of the 16 NDFU center survey respondents, n=7 (44%) reported seeing fewer than 10 infants for developmental follow-up per month, while only one clinic reported seeing more than 40 NICU graduates per month. Among the four responding NDFU clinics, assessments performed included medical (n=2, 50%) and developmental assessments [(motor (n=4, 100%), cognitive (n=3, 75%), speech and language (n=4, 100%)], along with occupational therapy (n=3, 75%), physical therapy (n=3, 75%), audiology (n=1, 25%), nutrition (n=2, 50%), and feeding/swallow evaluations (n=2, 50%). Of the 12 responding EI programs, none of the respondents reported conducting medical assessments; however, EI program respondents did perform motor (n=11, 92%), cognitive (n=11, 92%), and speech a language (n=10, 83%) assessments, as well as occupational therapy (n=10, 83%), physical therapy (n=9, 75%), and clinical feeding evaluations (n=6, 50%). They also reported completing audiology (n=2, 17%) and vision (n=5, 42%) assessments. Social work support was available in most ND clinics (n=3, 75%) and in half of EI programs (n=6, 50%). None (0%) of the NDFU programs responding reported performing or documenting specific screening for social determinants of health (SDOH).

**Figure 2:**
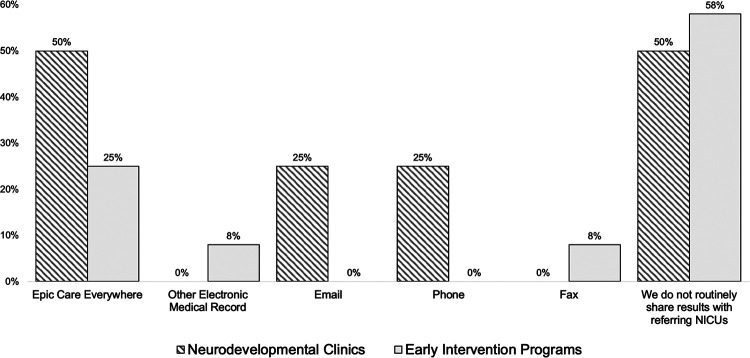
Methods Used by NDFU Centers to Share Developmental Testing Results with Referring NICUs^1^ Grey bars indicate methods of communication used by Early Intervention programs and hatched bars indicate methods of communication used by Neurodevelopmental Clinics. ^1^Note that 0 (0%) of NICU survey respondents reported receiving information from NDFU programs regarding developmental testing for referred infants.

We explored the methods NDFU centers used to share the results of post-discharge developmental testing and infant outcomes with referring NICUs (Figure 2). While three (25%) EI programs and two (50%) NDFU clinics reported using the electronic health record to share results, one (8%) EI program used clinic-to-clinic messaging, and one NDFU clinic (25%) used email or phone to communicate with referring NICUs. Notably, 100% of the surveyed NICU providers reported not receiving communication from NDFU clinics or EI programs with results of post-discharge neurodevelopmental testing or infant outcomes. (Figure 2)

Three-quarters of responding centers reported having a method to track no-show appointments. Of those tracking no-shows, nine programs (75%) had a no-show rate below 25%, while two (17%) had a no-show rate between 26% and 50%. Notably, some EI programs track no-show rates based on program-wide data rather than specifically to patients following up post-NICU discharge affecting the accuracy of reported attrition. For instance, one EI program respondent mentions, “This percentage rate…is our total no-show cancel rate of all of our ESIT services and not specific to NICU follow-up services.” Among all NDFU center survey respondents, most (n=12, 75%) offered some form of telehealth.

### Barriers and Facilitators for Neurodevelopmental Follow-up

**Figure 3:**
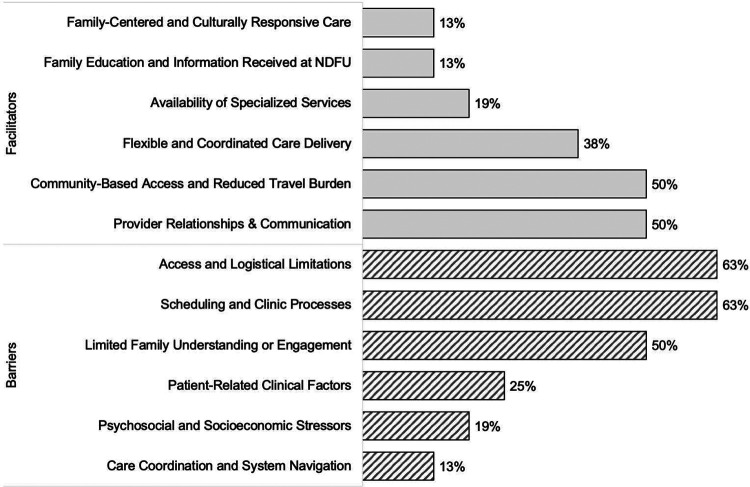
Thematic Analysis of Barriers and Facilitators to Neurodevelopmental Follow-Up Frequency of barrier and facilitator themes (defined in Table 2) were analyzed from open-ended survey questions of NDFU center providers. Gray bars indicate facilitators, and hatched bars indicate barriers to NDFU

Among NDFU center survey respondents who provided free-text comments, six barrier themes and six facilitator themes were identified through inductive content analysis (Figure 3, Table 2). Although several reported barriers were interrelated, they were grouped into themes based on the predominant way in which they interfered with attendance. The most frequently cited barrier themes to NDFU attendance were *logistical limitations* (n=10, 63%) and *scheduling processes* (n=10, 63%). Logistical limitations included competing caregiver responsibilities (n=6, 38%), transportation challenges (n=5, 31%), and housing instability or relocation (n=2, 13%). Scheduling processes included scheduling conflicts (n=4, 25%), forgotten appointments (n=3, 19%), long waitlists (n=1, 6%), or lack of patient information (n=1, 6%). Half of respondents (n=8, 50%) reported limited family understanding or engagement as a barrier to NDFU attendance, reflecting concerns about caregiver readiness (n=2, 13%), language barriers (n=1, 6%), or uncertainty about the purpose or importance of NDFU (n=7, 44%). Less commonly identified barriers included patient-related clinical factors (e.g., patient or caregiver illness, or rehospitalization) (n=4, 25%), psychosocial and socioeconomic stressors (n=3, 19%), and care coordination and system navigation challenges (n=2, 13%).

The most frequently identified facilitator themes of NDFU attendance were *provider relationships and communication* (n=8, 50%) and *community-based access and reduced travel burden* (n=8, 50%) (Table 2). Responses within the provider relationships and communication theme emphasized supportive provider interactions (n=7, 44%) and accessible communication (n=1, 6%). Community-based access and reduced travel burden included home-based services (n=5, 31%), telehealth availability (n=2, 13%), NDFU center location (n=2, 13%), and transportation assistance (n=1, 6%). Additional facilitator themes included flexible and coordinated care delivery (n=6, 38%) and availability of specialized services (n=3, 19%). Less commonly cited facilitator themes were family education and information received at NDFU visits (n=2, 13%) and family-centered and culturally responsive care (n=2, 13%). Together, these findings demonstrate that logistical access and clinic workflow factors were the most prominent barriers, whereas relational aspects of care and proximity of services were the most frequently described facilitators.

## Discussion

This survey of regional NICUs and NDFU centers is the first study to examine current practices, potential barriers, and facilitators of neurodevelopmental assessment and follow-up for high-risk infants across Washington state. This study’s findings revealed that 73.3% of surveyed Washington state NICUs do *not* perform standardized neurodevelopmental evaluations of neonates during their NICU hospitalization. While the criteria for NDFU referral are similar, referral placement and scheduling practices differ across programs, adding to confusion and fragmentation of care for high-risk infants. NDFU centers (including NDFU clinics and EI programs) report variable patient volumes, developmental assessments performed, and availability of telehealth services. In addition to potentially creating disparities in care, these differences may complicate longitudinal evaluation of high-risk infants who relocate or change developmental care providers.

### Neonatal and Neurodevelopmental Follow-up Care in Washington State

In 2024, one in eleven, or 9.0% of infants born in Washington, were premature (<37 weeks’ gestation), resulting in roughly 7,600 NICU admissions annually (49), not including term infants with both pre- and postnatal diagnoses of complex congenital disorders or birth-related trauma requiring NICU-level care. The regionalization of perinatal and neonatal care in Washington state generally concentrates services in urban economic centers, creating challenges for families who must travel from the state’s vast rural regions to large academic centers to access care. This is likely a commonality shared with other states and settings throughout the US and globally.

### Inter-program Communication: NICU and Neurodevelopmental Follow-up Centers

This analysis highlights opportunities to improve communication between NDFU centers and the NICUs who refer patients to them. We identified significant variation among institutions regarding which NICU provider or staff member places NDFU referrals, including discharge coordinators, care coordinators, medical staff, infant therapy staff, and social workers. Although NICU providers who place referrals may be alerted that a child is seen at an NDFU center, results are not tracked in a central data repository, and while approximately 50% of NDFU center respondents report sharing evaluation data with referring NICUs, *0% of the NICU providers stated that they routinely receive data on their referred patients*. These findings highlight a striking disconnect between the NDFU centers and the referring NICUs, elucidating an opportunity for enhancing communication between institutions.

The current lack of communication between institutions may limit the ability to conduct clinical audits, quality improvement initiatives, or research. Given the emphasis on regionalizing subspecialty neonatal and perinatal care in Washington state, there is a unique opportunity for broader institutional collaboration to track outcomes in these areas. Establishing a large regional database to which NICUs and NDFU programs submit de-identified data, similar to the California Perinatal Quality Care Collaborative (29) may play a broader role in tracking outcomes as process changes and advances in neonatal research continue to evolve.

### Practical and Logistical Considerations for NDFU Attendance

Given resource constraints that limit the expansion of NDFU appointments, strategies must be proposed to ensure that infants at the highest risk of NDI maintain timely access to evaluation and therapeutic services. The use of neurodevelopmental assessments during NICU admission, as part of early detection pathways, has led to earlier diagnosis of cerebral palsy (30) and earlier access to early intervention services, which may improve long-term functioning (31). Early detection and risk identification of NDI during NICU hospitalization using predictive models for risk stratification (32,33), may help shape referral pathways, and determine appropriate appointment timing, frequency, and modality for follow-up and early intervention.

While some aspects of follow-up care, such as hearing exams, must be conducted in person, some neurodevelopmental screening assessments can be conducted remotely via telehealth, potentially improving access to neurodevelopmental follow-up care. A large retrospective study of 55 Federally Qualified Health Centers (FQHCs) in Texas found that when appointments were available via telehealth rather than in person, the odds of a missed appointment were reduced by 13% (34). Moreover, a local investigation of pediatric neurology patients in Washington state during and after the conclusion of the COVID-19 pandemic, found that patients from rural areas of the state had far greater odds of utilizing telehealth even after in-person appointments could be rescheduled (35). During telehealth visits, experts may guide families in conducting certain developmental assessments of their child (36,37). This presents a valuable opportunity to engage families in care as parents learn how to observe and interpret their child’s movement and behavior(12,36,38). Finally, there is evidence that evaluating an infant in their home environment may be more reliable than an evaluation done in an unfamiliar exam room (39). Given the existing telehealth infrastructure through a majority of regional NDFU centers, and our finding that 75% of NDFU centers surveyed utilize telehealth services, more emphasis on utilizing this technology may improve access to care and directly overcome several key identified barriers to NDFU including concerns about competing caregiver responsibilities, limited means of transportation, and rising costs of childcare for other children in the household.

Social disparities have been found to be associated with reduced rates of NDFU. (23) In a large, population-based investigation in California, sociodemographic variables such as lower maternal education and single-parent households with reduced resources were associated with lower rates of neonatal follow-up before 12 months of corrected age (40). Given that no NDFU centers surveyed formally evaluate aspects of SDOH, this also highlights a potential opportunity for a simple screening assessment at intake or during appointment scheduling to help identify families at high risk of becoming lost to follow-up. For example, in response to these survey results, clinics at the Institute for Human Development and Disability (IHDD) at the University of Washington have initiated quality improvement work to improve SDOH screening documentation paired with immediate assistance with resources for needy families. Implementation of these processes on a larger scale would necessitate NDFUs to be resourced to support targeted interventions to improve access to care.

### Anticipatory Guidance

Our thematic analysis identified six prominent barriers, including logistical limitations, scheduling processes, and limited family understanding or engagement, which emerged as the most significant factors perceived by NDFU providers to negatively impact attendance. Concerns regarding the caregiver responsibilities, lack of transportation, affordable childcare, missed workdays to attend clinic visits, language barriers, and other family stressors have been described in other investigations, as has the concept that families often do not understand the rationale for NDFU (41,42). A modified Delphi study performed by Miller et al (2025) explored interventions to strengthen the NICU-to-home transition and identified “anticipatory guidance” for families, including discussions of important referrals such as NDFU and EI, as a crucial component of NICU discharge. However, finding an appropriate time to disclose concerns regarding an infant’s development that takes into consideration parental stress and anxiety surrounding uncertain prognoses is challenging (43–47).

We explored how families receive information about their infants’ developmental progress before NICU discharge, and how that information is typically disclosed. From our surveys of NICU providers, we found that most developmental testing results are shared directly with families by the NICU’s physical, occupational, or speech therapists, while 4 (27%) of the NICUs surveyed hold interdisciplinary meetings between providers and families before discharge to discuss testing, such as imaging or developmental screens. Unsurprisingly, respondents felt that parents’ understanding of the importance of NDFU can serve as a key facilitator to attendance. Furthermore, the strong relationships that families build with their therapists and NDFU providers were seen as beneficial.

Prior studies have similarly shown that family education initiatives are associated with improved follow-up attendance rates (48). Thus, by combining anticipatory guidance and family education prior to NICU discharge with the availability of home-based services and strategic adoption of telehealth after NICU discharge, paired with improved screening for perinatal mood and anxiety disorders and social determinants of health in both the NICU and NDFU periods, we may improve access to follow-up care and overcome systemic barriers to obtaining important screening tests for developmental delay in this vulnerable population. Furthermore, the development of a regional neonatal-perinatal database in WA state may bridge the gap in communication between academic and community NICUs and the outcomes of the infants that they serve.

## Strengths and Limitations

A main strength of this study is the high response rate, with correspondence occurring between all identified NICUs and NDFU/EI centers in WA state. Also, the collaboration with the Washington state Department of Health and the Perinatal Regional Nurses enhanced the ability to reach almost all NICUs and NDFU/EI centers in WA state. Additionally, our study is a contemporary cohort, making the findings generalizable to the current time. As a survey-based study, our design is subject to response bias, although our overall response rates were robust (i.e., 94% NICU providers, 84% NDFU center providers), which minimizes this concern.

However, our study has several limitations. One limitation includes relying on provider-reported survey data, which may be subject to recall bias and reflect individual respondents’ perspectives rather than institutional practice. Consequently, reported practices, particularly regarding referral, scheduling, and follow-up processes, may not fully reflect actual implementation in clinical workflows across institutions. While providers are well-positioned to identify systems-level processes and challenges, engagement in neurodevelopmental follow-up is also shaped by family experiences, preferences, and social determinants, which were not directly captured in this study. As a result, systems-level barriers and facilitators may be overrepresented relative to family-level determinants. While this study is the first assessment of NDFU practices across WA state, its findings may not generalize to other geographic regions with differing healthcare infrastructures, referral pathways, or access to follow-up services.

## Conclusion

This state-wide evaluation of NICU and NDFU programs identifies key opportunities to strengthen NDFU systems for high-risk infants. The variation in type and timing of screenings, assessments and support both within NICUs as well as in NDFU/EI centers may contribute to challenges in improving overall childhood health. Standardization of approaches may offer the benefit of statewide quality improvement opportunities. Barriers to returning for NDFU among high-risk families include limited access to clinics, difficulties in scheduling, and limited family understanding or engagement. Prominent facilitators include enhanced provider relationships and communication, as well as the flexibility of community- or home-based services. A lack of communication between NDFU programs on referred children’s outcomes and the referring NICUs highlights the need for a state-wide database or other methods to overcome this notable barrier for providers. Future research incorporating family perspectives, including qualitative interviews with families who do and do not attend NDFU, is a critical next step to inform the development of comprehensive, patient- and family-centered strategies to improve engagement in NDFU. These findings are crucial for enacting and implementing meaningful efforts to ultimately improve the care of high-risk newborns to help ensure that all children can live to their fullest potential.

## Supplementary Material

This is a list of supplementary files associated with this preprint. Click to download.

- NDFUcenterprovidersurvey.pdf
- NICUprovidersurvey.pdf

## Figures and Tables

**Table 1:** Characteristics of Surveyed Neonatal Intensive Care Unit (NICU) and Neurodevelopmental Follow-up (NDFU) Center Providers and Their Practice Settings

NICU Provider Survey (N=15)	n (%)	Criteria for NDFU Referral	n (%)
**Level of NICU**		Birth weight^1^	14 (93)
Level III	10 (67)	Gestational Age^2^	14 (93)
Level IV	5 (33)	Neonatal Opiate Withdrawal Syndrome	13 (87)
**Role of Respondent**		Hypoxic Ischemic Encephalopathy	12 (80)
Medical Director	6 (40)	Seizures	10 (67)
Nurse Manager	5 (33)	Cardiac Surgery	6 (40)
Case Manager	1 (7)	Non-Cardiac Surgery	5 (33)
Discharge Coordinator	1 (7)	Extra Corporeal Membrane Oxygenation	5 (33)
Lactation Consultant	1 (7)	Genetic Diagnosis	3 (20)
Therapy Services Manager	1 (7)	Perinatal Brain Injury	1 (7)
		Hyperbilirubinemia at Exchange	1 (7)
		Apgar <5 at 5 min	1 (7)
NDFU Center Provider Survey (N=16)	n (%)	NDFU Center Characteristics	n (%)
**Type of NDFU Center**		**Number of Patients Seen per Month**	
Early Intervention Program	12 (75)	Less than 10	7 (44)
Neurodevelopmental Clinic	4 (25)	10–20	5 (31)
**Role of Respondent**		21–30	2 (13)
Director	8 (50)	31–40	1 (6)
Program Manager	3 (19)	More than 40	1 (6)
Lead Therapist	3 (19)	**Clinic Utilizes Telehealth**	12 (75)
Care Manager	2 (13)	**Clinic Tracks No Shows**	12 (75)
		***% No Show at First Visit***^3^	
		0–25%	9 (75)
		26–50%	2 (17)
		Unknown	1 (8)
		***% No Show at 12-Month Visit***^3^	
		0–25%	6 (50)
		26–50%	1 (8)
		Unknown	5 (42)

1 NICU providers reported examples of birthweight criteria to include: ≤1500g at birth, Intrauterine Growth Restriction and Small for Gestational Age (<10%’tile).

2 Most gestational age criteria included infants <32 weeks’ gestation at birth, though the upper gestational age cut-off ranged from 30–37 weeks’ gestation.

3 Only NDFU center providers indicating their clinic tracked no-shows (N=12) were surveyed about percentages of no-shows at initial and 12-month visits.

**Table 2:** Defined Themes of Perceived Barriers and Facilitators to Neurodevelopmental Follow-Up

Barrier Theme	Definition	Facilitator Theme	Definition
**Care Coordination and System Navigation**	Challenges related to coordinating NDFU care and navigating the healthcare system, including gaps in coordination, unclear processes, and difficulty appropriate resources.	**Availability of Specialized Services**	Presence and accessibility of specialized clinical or therapeutic services within NDFU, including providers with specific areas of expertise such as infant mental health or specialized therapies.
**Limited Family Understanding or Engagement**	Family-level factors related to limited understanding of or engagement with NDFU, including unclear concerns, lack of readiness or interest, language barriers, and confusion about the purpose or importance of NDFU.	**Community-Based Access & Reduced Travel Burden**	Structural and operational features that increase access to care, including community-based NDFU center locations, home-based services, telehealth availability, and transportation assistance that reduce travel burden for follow-up.
**Logistical Limitations**	Factors that limit families' ability to access or attend NDFU, including transportation, time constraints, caregiver responsibilities, caregiver illness, housing instability, and competing life demands.	**Family-Centered and Culturally Responsive Care**	Care practices that actively involve families in care and adapt to families' cultural and linguistic contexts.
**Patient-Related Clinical Factors**	Acute or ongoing illness of the patient, including rehospitalization or post-discharge complications, that interferes with attendance or participation in NDFU services.	**Family Education and Information Received at NDFU**	Families receiving, understanding, and valuing information about their child's care and services at NDFU, including explanations of why and how they are involved in programs or therapies.
**Psychosocial and Socioeconomic Stressors**	Social, emotional, and economic stressors that affect families' capacity to engage in NDFU, including family dynamics, financial strain, psychosocial distress, and prior negative healthcare experiences.	**Flexible and Coordinated Care Delivery**	NDFU center structures and processes that promote coordinated, continuous, and adaptable access to services, including flexible scheduling, streamlined intake, and goal-oriented care-planning.
**Scheduling Processes**	Clinic- and system-level processes related to appointment scheduling that affect families' ability to attend NDFU visits, including scheduling conflicts, long waitlists, forgotten appointments, and outreach challenges.	**Provider Relationships and Communication**	Relational and communication aspects of care, including supportive provider interactions, consistent therapeutic relationships, and accessible communication between families and care team members.

Barrier and facilitator themes were defined for open-ended survey questions of NDFU center providers.

## Data Availability

The data that support the findings of analysis may be made available at reasonable request.
